# Association of rural-urban place of residence with adequate antenatal care visit in Bangladesh

**DOI:** 10.1371/journal.pgph.0002528

**Published:** 2023-10-25

**Authors:** Gulam Muhammed Al Kibria, Jannatun Nayeem

**Affiliations:** 1 Johns Hopkins University Bloomberg School of Public Health, Baltimore, MD, United States of America; 2 Comilla Medical College, Comilla, Bangladesh; Institution of Clinical Sciences Lund, Lund University, SWEDEN

## Abstract

Adequate antenatal care (ANC) is crucial to reduce maternal/neonatal deaths, but the proportion of mothers with appropriate ANC is lower in most low- and middle-income countries (LMICs) than in high-income countries. Furthermore, in many LMICs, including Bangladesh, there are intra-country disparities, and rural regions have substantially lower adequate ANC than urban regions. In this cross-sectional study, we performed secondary analyses on Bangladesh Demographic and Health Survey 2017–18 data to examine the rural-urban differences in adequate initiation, number, and quality of ANC. Women of reproductive age (i.e., 15-49-year-olds) with at least one birth in the three years before the survey were included. After reporting the prevalence of adequate ANC by rural-urban place, simple and multivariable logistic regression analyses were performed to examine the association. Among 4974 women, 1331 and 3643 were from urban rural regions, respectively. The proportions of mothers who initiated the visits in the first trimester, had at least four ANC visits, and had quality ANC were 47.5% (95% confidence interval (CI): 44.6–50.3), 59.0% (95% CI: 56.3–61.8), and 27.1% (95% CI: 24.6–29.6) in urban regions, and 33.4% (95% CI: 31.6–35.1), 42.8% (95% CI: 41.0–44.7), and 14.5% (95% CI: 13.1–15.8) in rural regions, respectively. These differences in ANC utilization were observed regardless of most sociodemographic and socioeconomic characteristics. After adjusting for sociodemographic and socioeconomic characteristics, compared to urban mothers, rural mothers had lower odds of at least four ANC visits (adjusted odds ratio (AOR): 0.77, 95% CI: 0.65–0.91) and quality ANC (AOR: 0.79, 95% CI: 0.65–0.97) but the odds for timely initiation (AOR: 0.85, 95% CI: 0.73–1.01) was not significant. Findings of this study showed significant disparities between rural and urban regions regarding appropriate ANC coverage, and the importance of improving ANC coverage among some sociodemographic groups to reduce these disparities, especially among mothers with low socioeconomic status.

## Introduction

Antenatal care (ANC) refers to the care provided during pregnancy to ensure optimal well-being for mothers and newborns [1–3]. Its purpose is to identify pregnancy-related risks or conditions and offer health education [3, 4]. It is considered the initial step in the continuum of care for pregnancies [1–3]. During the past couple of decades, the numbers of maternal and childhood deaths have declined all over the world. These numbers, however, remain high in many low- and middle-income countries (LMICs), including Bangladesh [5, 6]. This nation is now struggling to reduce maternal and childhood deaths. As per the estimates of the ‘Trends in Maternal Mortality’ report, the maternal mortality ratio (MMR) in Bangladesh was 173 per 100,000 live births in 2017 [6]. According to the Bangladesh Demographic and Health Survey (BDHS) 2017–18, the neonatal mortality rate (NMR) was 30 per 1,000 live births [7]. It should be noted that the primary reasons behind maternal and neonatal deaths can be prevented or effectively treated using currently available and inexpensive interventions [1–3]. For example, severe bleeding, infection, high blood pressure (such as pre-eclampsia and eclampsia), and delivery complications are the leading causes of maternal deaths [6]. Among newborns, prematurity/low birth weight, sepsis (i.e., infections), and perinatal asphyxia are the main causes of mortality [8]. To expand the implementation of these interventions and reduce deaths from the aforementioned causes, adequate ANC has been recommended as the primary approach [3, 9, 10].

The World Health Organization (WHO) advises beginning ANC in the first trimester of pregnancy. They also recommend a minimum of eight ANC contacts [3]. However, the government of Bangladesh currently recommends at least four ANC visits [11]. The quality of ANC is crucial and includes at least four visits, with at least one conducted by a medically trained provider, along with screenings and information like blood pressure measurements [7]. Despite the importance of ANC in reducing pregnancy-related deaths, the rates of timely initiation, at least four visits, at least eight visits, and quality ANC are low in Bangladesh, represented by 37%, 47%, 11%, and 18%, respectively. Furthermore, there are disparities between rural and urban regions in this country. In rural areas, the rates of timely initiation, at least four visits, at least eight visits, and quality ANC are low, represented by 33%, 43%, 9%, and 14%, respectively. However, in urban areas, these rates are higher, represented by 47%, 59%, 17%, and 27%, respectively [7]. About two-thirds of Bangladeshi people live in rural regions [7, 12]. Without improving the ANC uptake in these regions, it will be a big challenge to increase ANC uptake in the overall country.

Multiple previous studies from Bangladesh and other countries also reported the socioeconomic disparities associated with ANC, and women with lower socioeconomic status were less likely to receive adequate ANC than those with higher socioeconomic status [9, 13–16]. While previous studies have identified factors associated with timely initiation, the number of visits, and the quality of ANC in Bangladesh, there has been limited investigation into whether these relationships differ based on rural-urban residence, especially after adjusting for sociodemographic and socioeconomic factors that can also impact the differences in utilization. It is also unknown whether the differences in ANC uptake remain similar following stratification by rural-urban regions. We aimed to address these gaps in knowledge and examined the disparities between rural and urban areas regarding the initiation, number of visits, and quality of ANC in Bangladesh. The findings of this study will be valuable for researchers and policymakers in designing and implementing effective programs and policies to promote ANC services in this country.

## Methods

### Study design and settings

In this cross-sectional study, we conducted secondary analyses on the latest BDHS 2017–18 data [7]. The survey aims to collect indicators related to maternal and child health, including maternal healthcare utilization. It covered rural and urban regions of all administrative divisions. Mitra and Associates, a private research group in Bangladesh, conducted the survey. Interviews were conducted by 200 trained data collectors between October 2017 and March 2018. The study included women of reproductive age who were currently married and had at least one pregnancy history. Bangladesh is a country located in South Asia, with an estimated population of 170 million, and an area of 55,000 square miles. The predominant religion is Islam, with Hinduism and other religions being practiced by a smaller percentage of the population [7].

### Sample coverage

The survey began by creating a sample frame, using the housing and population census of 2011, which included a list of enumeration areas (EA) and was designed to provide separate estimates for rural and urban regions and each administrative division [7, 12]. Households were selected in two stages. A total of 425 EAs were selected from rural regions, and 250 EAs were selected from urban regions. The EAs were selected based on probability proportional to their size. From each EA, 30 households were selected randomly, and the survey report, methodology, sample size calculation, and questionnaires are available online. The response rate was 98%, resulting in a total of 20,127 women being interviewed from 20,160 households. The survey focused on the history of the last pregnancy within the past three years, to minimize recall bias and have the most recent data [7].

### Ethics statement

Ethical approval of BDHS 2017–18 received was obtained from the institutional review boards (IRBs) at ICF International (IRB: FWA00000845) and the Bangladesh Medical Research Council (IRB: BMRC/NREC/2016–2019/324). Respondents provided verbal informed consent to participate in the survey, if a participant was below 18 years of age, an adult household member provided consent. Electronic approval for using the datasets was obtained from DHS program.

### Outcomes

This study evaluated three outcomes related to the recommended timing, number, and quality of ANC: (a) appropriate timing of initiation of ANC, defined as starting ANC during the first trimester of pregnancy; (b) adequate number (i.e., at least four) of ANC visits, based on the recommendation by the Government of Bangladesh; and (c) quality of ANC, defined as receiving at least four ANC visits, with at least one of these visits conducted by a medically trained provider, which includes four basic ANC components like body weight measurement, blood pressure measurement, urine and blood sample collection and testing, and dissemination of information about danger signs during pregnancy. Medically trained providers included qualified doctors, nurses, midwives, paramedics, family welfare visitors, community skilled birth attendants, and sub-assistant community medical officers [7].

### Exposures

This study used a selection of variables as potential determinants of the recommended timing, number, and quality of ANC, based on published studies, scientific plausibility, and the BDHS 2017–18 dataset. These variables included maternal age, parity, women’s and husband’s education, current work status, religion, household wealth status, and rural-urban residence and division. Maternal age was grouped into three categories: 15–24, 25–34, and 35–49 years. Parity was categorized as either ‘first pregnancy’, or ‘second or more’. Women were asked about their education and that of their husbands. Education was divided into four categories: no formal education, primary (i.e., 1 to 5 school years), secondary (i.e., 6 to 10 school years), and college or above (i.e., 11 or more school years). Respondents also provided information about their current work status, their place of residence (i.e., rural or urban), and the administrative division in which they lived. Bangladesh had eight divisions in 2017–2018: Dhaka, Chittagong, Rajshahi, Khulna, Barishal, Rangpur, Sylhet, and Mymensingh. Household wealth was divided into quintiles (i.e., poorest, poorer, middle, richer, and richest) based on a household wealth index score, which was calculated using principal component analysis of factors such as the materials used to construct the household, access to water and sanitation facilities, electricity, and household possessions [7].

### Statistical analyses

The first step of the analysis was to describe the basic sociodemographic/socioeconomic characteristics of the respondents by rural-urban place of residence, using weighted frequencies and percentages for the variables. The characteristics of the respondents were compared across outcomes using chi-squared tests. The study also reported the prevalence of all three outcomes, along with 95% confidence intervals (CIs) by rural-urban places. Lastly, we used unadjusted and adjusted multilevel logistic regression to investigate the association of all three outcomes with rural-urban residence. Variables that were found to be associated with the outcomes in the unadjusted regression were included in the multivariable (i.e., adjusted) model. The study reported both unadjusted and adjusted odds ratios (ORs), along with the 95% CIs. We also checked for multicollinearity using variance inflation factors. The analysis was performed using SAS software, taking into account the hierarchical nature of the BDHS dataset and using the provided sample weights.

## Results

Among 4974 included mothers, 1333 were from urban regions and 4631 were from rural regions (Table 1). A majority were younger than 25 years, at 53.0% (n = 2638). About 37.9% were primi. The proportion of mothers with a college education was higher in urban regions than in rural regions, at 24.4% (n = 325) and 14.6% (n = 533), respectively. Similarly, urban regions had a higher proportion of mothers with college-educated husbands, or richer household wealth quintiles than rural regions. Most of the mothers were Muslims and the highest proportion of mothers was from Dhaka division, at 91.8% (n = 4568) and 25.6% (n = 1274), respectively.

**Table 1:** Sample characteristics by place of residence.

**Variable**		**Overall**	**Urban**	**Rural**	**p-value**
		**(n = 4974)**	**(n = 1333)**	**(n = 3641)**	
Age (in year)	15–24	53.0 (2638)	50.0 (666)	54.2 (1972)	0.032
	25–34	41.1 (2044)	43.6 (582)	40.2 (1462)	
	35–49	5.9 (292)	6.4 (85)	5.7 (207)	
Parity	Primi	37.9 (1887)	40.6 (542)	37.0 (1345)	0.018
	≥2	62.1 (3087)	59.4 (791)	63.0 (2296)	
Birth Interval	< = 2-year	338 (6.8)	77 (5.8)	261 (7.2)	0.023
	>2 year	2733 (55.1)	708 (53.4)	2025 (55.8)	
	Primi	1887 (38.1)	542 (40.8)	1345 (37.0)	
Women’s education level	No education	6.2 (309)	6.4 (85)	6.2 (224)	<0.001
	Primary	27.5 (1370)	25.1 (335)	28.4 (1035)	
	Secondary	49.0 (2437)	44.1 (588)	50.8 (1849)	
	College/Above	17.2 (858)	24.4 (325)	14.6 (533)	
Husband’s education level	No education	13.7 (680)	9.8 (131)	15.1 (549)	<0.001
	Primary	33.7 (1678)	29.6 (394)	35.3 (1284)	
	Secondary	34.1 (1696)	34.4 (459)	34.0 (1237)	
	College/Above	18.5 (920)	26.2 (349)	15.7 (571)	
Respondent work status	No	62.9 (3129)	71.6 (954)	59.7 (2175)	<0.001
	Yes	37.1 (1845)	28.4 (379)	40.3 (1466)	
Religion	Muslim	91.8 (4568)	92.8 (1238)	91.5 (3330)	0.12
	Other	8.2 (406)	7.2 (95)	8.5 (311)	
Wealth quintile	Poorest	20.7 (1027)	8.7 (116)	25.0 (911)	<0.001
	Poorer	20.4 (1013)	6.4 (85)	25.5 (928)	
	Middle	19.1 (948)	11.8 (158)	21.7 (790)	
	Richer	20.3 (1011)	27.5 (367)	17.7 (644)	
	Richest	19.6 (974)	45.6 (607)	10.1 (367)	
Division of residence	Dhaka	25.6 (1274)	46.9 (625)	17.8 (649)	<0.001
	Chittagong	21.2 (1054)	18.6 (247)	22.2 (807)	
	Barisal	5.7 (282)	3.6 (47)	6.5 (235)	
	Khulna	9.1 (453)	7.8 (104)	9.6 (349)	
	Mymensingh	8.5 (424)	5.5 (74)	9.6 (350)	
	Rajshahi	11.6 (579)	7. 7(103)	13.1 (476)	
	Rangpur	10.6 (528)	5.6 (75)	12.5 (453)	
	Sylhet	7.6 (378)	4.3 (57)	8.8 (321)	

Tables 2–4 present the prevalence of timely initiation, at least four, and quality ANC, respectively, by background characteristics in rural and urban regions. The proportion of mothers who initiated the visits in the first trimester, had at least four ANC visits, or quality ANC was 47.5% (95% CI: 44.6–50.3), 59.0% (95% CI: 56.3–61.8), and 27.1% (95% CI: 24.6–29.6) in urban regions; and 33.4% (95% CI: 31.6–35.1), 42.8% (95% CI: 41.0–44.7), and 14.5% (95% CI: 13.1–15.8) in rural regions; and 37.2% (95% CI: 35.7–38.7), 47.2% (95% CI: 45.6–48.7), and 17.9% (95% CI: 16.7–18.0) in the overall country, respectively. Regardless of most background characteristics, the overall prevalence of all 3 studied outcomes was higher in urban regions than rural regions. For instance, among 15-24-year-old mothers, the proportion of mothers with at least four ANC was 57.1% (95% CI: 53.1–61.1) in urban regions and 44.0% (95% CI: 41.5–46.5) in rural regions.

**Table 2:** Timely initiation of ANC visits overall and by place of residence.

Variable		Overall	Urban	Rural
Age (in year)	15–24	37.1 (35.0–39.1)	44.5 (40.6–48.5)	34.6 (32.1–37.0)
	25–34	38.3 (35.9–40.6)	51.3 (47.0–55.5)	33.1 (30.3–35.8)
	35–49	30.3 (24.6–36.0)	44.3 (33.6–55.0)	24.5 (18.0–31.1)
Parity	Primi	42.8 (40.3–45.3)	52.2 (47.7–56.6)	39.0 (36.0–42.0)
	≥2	33.7 (31.8–35.6)	44.2 (40.6–47.9)	30.1 (36.0–42.0)
Birth interval	< = 2-year	27.4 (22.3–32.6)	36.0 (24.9–47.0)	45.0 (41.1–48.9)
	>2-year	34.4 (32.4–36.4)	25.0 (19.2–30.7)	30.7 (28.4–33.0)
Women’s education level	No education	20.2 (15.2–25.1)	23.5 (13.8–33.2)	18.9 (13.2–24.6)
	Primary	26.1 (23.4–28.7)	30.0 (24.6–35.3)	24.8 (21.8–27.8)
	Secondary	37.3 (35.1–39.4)	46.2 (42.0–50.4)	34.5 (32.0–36.9)
	College/Above	60.7 (57.1–64.3)	74.2 (69.4–79.0)	52.5 (47.6–57.4)
Husband’s education level	No education	22.3 (18.9–25.7)	23.1 (15.6–30.7)	22.1 (18.3–25.9)
	Primary	27.6 (25.2–30.0)	28.6 (23.9–33.4)	27.3 (24.5–30.0)
	Secondary	39.7 (37.1–42.4)	50.5 (45.7–55.3)	35.7 (32.6–38.8)
	College/Above	60.9 (57.4–64.4)	73.8 (69.2–78.4)	53.0 (48.2–57.8)
Respondent work status	No	40.4 (38.4–42.3)	50.8 (47.4–54.2)	35.8 (33.5–38.2)
	Yes	31.7 (29.4–34.0)	39.1 (34.1–44.1)	29.8 (27.2–32.4)
Religion	Muslim	37.0 (35.4–38.5)	47.6 (44.7–50.5)	33.0 (31.2–34.8)
	Other	39.5 (34.3–44.7)	45.5 (35.7–55.3)	37.7 (31.6–43.8)
Wealth quintile	Poorest	22.1 (19.4–24.9)	20.3 (13.7–26.9)	22.4 (19.4–25.3)
	Poorer	30.2 (27.1–33.3)	29.7 (20.7–38.6)	30.2 (26.9–33.5)
	Middle	35.0 (31.5–38.5)	28.2 (21.6–34.8)	36.4 (32.4–40.3)
	Richer	38.4 (35.0–41.8)	36.2 (31.0–41.4)	39.7 (35.2–44.1)
	Richest	61.0 (57.6–64.5)	66.9 (62.9–71.0)	51.3 (45.3–57.3)
Division of residence	Dhaka	46.3 (42.6–50.0)	52.6 (47.7–57.5)	40.2 (34.8–45.6)
	Chittagong	31.4 (28.2–34.7)	35.3 (29.4–41.1)	30.3 (26.4–34.1)
	Barisal	30.4 (26.3–34.6)	39.4 (31.6–47.2)	28.6 (23.9–33.3)
	Khulna	38.8 (34.3–43.2)	48.7 (41.4–56.0)	35.8 (30.5–41.1)
	Mymensingh	36.1 (32.0–40.7)	43.7 (33.8–53.6)	34.5 (30.0–39.0)
	Rajshahi	31.9 (27.8–36.1)	46.1 (38.0–54.2)	28.8 (24.2–33.5)
	Rangpur	34.2 (30.0–38.3)	54.6 (46.8–62.3)	30.8 (26.2–35.5)
	Sylhet	38.8 (34.9–42.6)	46.1 (39.1–53.2)	37.5 (33.1–41.8)
Overall		37.2 (35.7–38.7)	47.5 (44.6–50.3)	33.4 (31.6–35.1)

**Table 3:** Prevalence of at least ANC visits overall and by place of residence.

Variable		Overall	Urban	Rural
Age (in year)	15–24	47.3 (45.1–49.4)	57.1 (53.1–61.1)	44.0 (41.5–46.5)
	25–34	48.2 (45.8–50.6)	62.1 (58.0–66.2)	42.7 (39.8–45.6)
	35–49	38.9 (32.8–45.0)	53.4 (42.7–64.1)	33.0 (25.8–40.1)
Parity	Primi	54.1 (51.6–56.6)	64.7 (60.4–69.0)	49.9 (46.8–52.9)
	≥2	42.9 (41.0–44.9)	55.2 (51.5–58.8)	38.7 (36.4–41.0)
Birth interval	< = 2-year	36.2 (30.6–41.7)	44.6 (33.2–56.1)	56.0 (52.2–59.9)
	>2-year	43.7 (41.6–45.8)	33.7 (27.4–40.0)	39.4 (37.0–41.8)
Women’s education level	No education	20.2 (15.3–25.1)	25.6 (15.6–35.6)	18.1 (12.5–23.7)
	Primary	34.0 (31.2–36.8)	41.8 (36.1–47.5)	31.5 (28.3–34.7)
	Secondary	50.3 (48.0–52.5)	62.9 (58.8–67.0)	46.3 (43.7–48.9)
	College/Above	69.1 (65.6–72.6)	78.8 (74.2–83.3)	63.3 (58.5–68.0)
Husband’s education level	No education	29.8 (26.0–33.5)	37.0 (28.3–45.7)	28.1 (23.9–32.2)
	Primary	37.2 (34.6–39.8)	47.1 (41.9–52.3)	34.1 (31.2–37.1)
	Secondary	50.6 (47.9–53.3)	58.8 (54.0–63.6)	47.5 (44.3–50.7)
	College/Above	71.9 (68.7–75.2)	81.1 (77.0–85.2)	66.3 (61.8–70.8)
Respondent work status	No	48.0 (46.0–49.9)	61.0 (57.7–64.3)	42.2 (39.8–44.7)
	Yes	45.9 (43.4–48.3)	54.2 (49.0–59.3)	43.7 (40.9–46.5)
Religion	Muslim	46.4 (44.8–48.0)	59.1 (56.2–62.0)	41.7 (39.8–43.6)
	Other	55.6 (50.4–60.9)	58.1 (48.4–67.9)	54.9 (48.7–61.1)
Wealth quintile	Poorest	30.9 (27.9–33.9)	29.1 (21.8–37.0)	31.1 (27.8–34.4)
	Poorer	36.7 (33.4–39.9)	41.8 (32.4–51.3)	36.2 (32.8–39.6)
	Middle	45.6 (42.0–49.3)	44.1 (36.5–51.8)	45.9 (41.8–50.0)
	Richer	51.8 (48.3–55.3)	48.8 (43.3–54.3)	53.5 (49.0–58.1)
	Richest	72.0 (68.8–75.1)	77.2 (73.6–80.9)	63.3 (57.6–69.0)
Division of residence	Dhaka	51.4 (47.7–55.1)	62.0 (57.2–66.8)	41.3 (35.9–46.7)
	Chittagong	38.7 (35.3–42.1)	48.5 (42.3–54.6)	35.7 (31.7–39.7)
	Barisal	37.6 (33.2–41.9)	51.0 (42.9–59.0)	34.9 (29.9–39.8)
	Khulna	57.8 (53.3–62.3)	68.6 (61.7–75.5)	54.6 (49.1–60.0)
	Mymensingh	45.2 (41.0–49.4)	53.1 (43.1–63.2)	43.5 (38.9–48.2)
	Rajshahi	48.0 (43.5–52.5)	64.2 (56.4–72.1)	44.4 ((39.3–49.6)
	Rangpur	59.6 (55.3–64.0)	72.1 (65.2–79.0)	57.6 (52.6–62.6)
	Sylhet	34.5 (30.7–38.2)	43.1 (36.1–50.1)	32.9 (28.7–37.2)
Overall		47.2 (45.6–48.7)	59.0 (56.3–61.8)	42.8 (41.0–44.7)

**Table 4:** Prevalence of quality ANC visits overall and by place of residence.

Variable		Overall	Urban	Rural
Age (in year)	15–24	16.6 (15.0–18.2)	22.9 (19.6–26.3)	14.4 (12.6–16.2)
	25–34	20.1 (18.2–22.1)	32.5 (28.5–36.5)	15.2 (13.1–17.3)
	35–49	13.3 (9.2–17.4)	23.3 (14.5–32.2)	9.2 (4.8–13.6)
Parity	Primi	15.7 (14.3–17.1)	29.9 (25.9–33.9)	17.9 (15.6–20.3)
	≥2	21.4 (19.3–23.4)	25.2 (22.1–28.4)	12.4 (10.9–14.0)
Birth interval	< = 2-year	12.4 (8.5–16.2)	21.1 (11.4–30.8)	25.3 (22.0–28.7)
	>2-year	16.0 (14.5–17.5)	9.8 (5.8–13.8)	12.7 (11.0–14.4)
Women’s education level	No education	5.7 (2.9–8.5)	11.3 (4.0–18.6)	3.5 (0.8–6.2)
	Primary	8.9 (7.2–10.5)	15.2 (11.1–19.4)	6.8 (5.1–8.6)
	Secondary	17.9 (16.2–19.6)	26.4 (22.7–30.1)	15.2 (13.3–17.1)
	College/Above	36.5 (32.9–40.0)	45.0 (39.4–50.6)	31.3 (26.8–35.8)
Husband’s education level	No education	8.0 (5.8–10.3)	14.1 (7.3–20.9)	6.6 (4.3–8.9)
	Primary	9.6 (8.1–11.2)	15.2 (11.5–18.9)	7.9 (22.1–30.6)
	Secondary	19.4 (17.3–21.6)	26.3 (22.1–30.6)	16.9 (14.5–19.3)
	College/Above	37.1 (33.7–40.6)	46.6 (41.2–52.0)	31.4 (26.9–35.8)
Respondent work status	No	19.3 (17.7–20.9)	28.1 (25.1–31.1)	15.5 (13.7–17.2)
	Yes	15.4 (13.6–17.1)	24.7 (20.3–29.1)	13.0 (11.1–14.8)
Religion	Muslim	17.6 (16.4–18.8)	27.3 (24.7–29.9)	14.0 (12.6–15.3)
	Other	20.9 (16.6–25.1)	25.5 (17.1–33.8)	19.4 (14.5–24.4)
Wealth quintile	Poorest	6.7 (5.1–8.2)	8.3 (4.1–12.6)	6.4 (4.8–8.1)
	Poorer	9.0 (7.2–10.9)	12.5 (6.3–18.6)	8.7 (6.8–10.7)
	Middle	15.7 (13.0–18.3)	12.3 (7.5–17.1)	16.4 (14.6–22.9)
	Richer	21.8 (18.9–24.7)	18.7 (14.6–22.9)	23.6 (19.7–27.4)
	Richest	36.8 (33.4–40.2)	41.7 (37.5–46.0)	28.7 (23.2–34.2)
Division of residence	Dhaka	22.9 (19.9–25.9)	30.2 (25.8–34.6)	15.9 (11.8–19.9)
	Chittagong	14.8 (12.4–17.2)	22.7 (17.7–27.8)	12.4 (9.6–15.1)
	Barisal	16.0 (12.6–19.4)	20.8 (14.0–27.6)	15.0 (11.2–18.8)
	Khulna	20.5 (16.9–24.1)	28.5 (22.0–35.1)	18.1 (13.9–22.4)
	Mymensingh	15.9 (12.9–18.9)	24.5 (16.7–32.3)	14.1 (10.8–17.3)
	Rajshahi	15.6 (12.4–18.8)	23.7 (16.9–30.5)	13.8 (10.2–17.4)
	Rangpur	19.5 (16.1–23.0)	32.4 (25.1–39.7)	17.4 (13.6–21.2)
	Sylhet	10.9 (8.5–13.2)	18.3 (12.9–23.6)	9.6 (7.0–12.2)
Overall		17.9 (16.7–18.0)	27.1 (24.6–29.6)	14.5 (13.1–15.8)

Table 5 shows the unadjusted and adjusted associations of place of residence with all three outcomes. The unadjusted odds were significantly lower for all outcomes. Although in the adjusted analysis, the odds for timely initiation (adjusted OR (AOR): 0.85, 95% CI: 0.73–1.01) were not significantly lower in rural regions than urban regions, the odds for at least four ANC visits (AOR: 0.77, 95% CI: 0.65–0.91) and quality ANC (AOR: 0.79, 95% CI: 0.65–0.97) were significantly lower.

**Table 5:** Association of place of residence with 3 outcomes.

Place of residence (Ref: Urban)		Initiated in first trimester	At least four ANC visits	Quality ANC visits
Rural	Unadjusted	0.55 (0.48-.0.64)	0.52 (0.45–0.60)	0.45 (0.38–0.54)
	Adjusted	0.85 (0.73–1.01)^1^	0.77 (0.65–0.91)^2^	0.79 (0.65–0.97)^1^

1. Adjusted for age, birth interval, parity, women’s education, husband’s education, work status, wealth status, and division of residence.

2. Adjusted for age, birth interval, parity, women’s education, husband’s education, work status, religion, wealth status, and division of residence.

## Discussion

In this study, we examined the association of rural-urban residence with adequate ANC (i.e., timely initiation, at least four visits, and quality ANC) and found that although a significantly lower proportion of mothers in rural regions received adequate numbers or quality of ANC, the association was not significant for initiation of ANC in the first trimester. These differences were observed regardless of most sociodemographic and socioeconomic characteristics.

When we compared the sample by rural-urban place of residence, we observed that the socioeconomic status of urban mothers was significantly higher than that of their rural counterparts. After adjusting for background characteristics, the lower magnitude of the rural-urban place of residence’s association suggests that socioeconomic status primarily contributed to this difference. Due to lower education levels, rural women or their husbands may be less aware than those in urban regions. Their lower wealth status may also prevent them from utilizing health care. It is important to reduce the socioeconomic inequalities between urban and rural regions. These differences may also contribute to other health outcomes (e.g., neonatal mortality rate), therefore, reducing the differences could reduce the disparities in health outcomes as well [7]. This study did not compare awareness about ANC; however, it raised the possibility that increasing awareness would improve ANC utilization in Bangladesh.

In addition to rural residence or socioeconomic status, there has been a significant difference in outcomes by division of residence. Previous studies reported socioeconomic differences between divisions of Bangladesh, and some divisions (e.g., Sylhet) had consistently low maternal health care utilization [7, 17]. Further research is required to examine ways to increase maternal and child health services.

Although our findings are not novel in the context of healthcare utilization/outcomes in Bangladesh, they have important policy implications. We showed that a large proportion of mothers do not receive adequate ANC care in this country, and rural mothers were less likely to receive this care than their urban counterparts. About two-thirds of the people in Bangladesh live in rural regions [12], and without reducing the differences found here, it would be hard to increase adequate ANC coverage. Apart from the socioeconomic differences mentioned above, there are several other barriers that could contribute to the differences in ANC utilization [9, 15, 18]. For instance, the distance from health facilities to homes and inadequate transport facilities in rural regions may prevent some women to receive adequate care [7, 19]. To increase the availability of maternal health services, the government has established multiple health centers (i.e., community clinics) [20, 21]. This was a fundamental step to increase health care utilization and coverage in a resource-limited country like Bangladesh. During the period of data collection (i.e., 2017–18) and the period of pregnancy history (i.e., up to 3 years prior to the survey), these community clinics were in their initial stages of implementation [7]. Future studies should examine how these community clinics have contributed to reducing rural-urban health disparities and the effectiveness of these clinics.

The strengths of our study include using a large sample covering rural and urban regions of all administrative divisions in Bangladesh, a high response rate, very low missing data, and collecting data using standardized and validated instruments. All these methods made the findings generalizable to the country [7].

However, this study has some limitations as well. For instance, we did not account for the availability or quality of care by region, which limited our ability to account for the variables contributing to the differences. This was a cross-sectional study, some of the variables may have changed since the pregnancy time, therefore, the observed association may not be causal. It included data of the past 3 years, this may cause some recall bias [7].

## Conclusion

This study addressed rural-urban differences in ANC utilization and how to increase it in Bangladesh. The disparities reported here may primarily result from socioeconomic disparities between the regions. Reducing these disparities would be challenging and may take a long time, improving ANC utilization would be crucial to improving maternal and neonatal health outcomes.

## Abbreviations

ANC: Antenatal care
BDHS: Bangladesh Demographic and Health Survey
CI: Confidence interval
LMIC: Low- and middle-income country
OR: Odds ratio
SE: Standard error
WHO: World Health Organization
